# Simultaneous Packaging of Two Different RNA Segments into an Influenza C Virus-like Particle Occurs Inefficiently

**DOI:** 10.3390/v17030350

**Published:** 2025-02-28

**Authors:** Yasushi Muraki

**Affiliations:** 1 Division of Infectious Diseases and Immunology, Department of Microbiology, School of Medicine, Iwate Medical University, 1-1-1 Idaidori, Yahaba 028-3694, Iwate, Japan; ymuraki@iwate-med.ac.jp; 2 Department of Infectious Diseases, Yamagata University Faculty of Medicine, 2-2-2 Iida-Nishi, Yamagata 990-9585, Japan

**Keywords:** influenza C virus, genome packaging, reverse genetics system, virus-like particles

## Abstract

Reverse genetics systems for influenza C virus encounter challenges due to the inefficient production of infectious virus particles. In the present study, we explored the underlying cause by assessing the efficiency of generating influenza C virus-like particles (C-VLPs) containing specific virus RNA (vRNA) segments. Using 293T cells transfected with plasmids encoding GFP- and DsRed2-vRNAs (each flanked by the non-coding regions of Segments 5 and 6, respectively), along with plasmids expressing virus proteins, we observed that C-VLPs containing a single vRNA segment were generated efficiently. However, the simultaneous packaging of two vRNA segments into a single C-VLP was less frequent, as demonstrated by flow cytometry and reverse-transcription PCR analyses. Statistical evaluations revealed a decreased efficiency of incorporating multiple vRNA segments into single particles, which likely contributes to the reduced production of infectious virus particles in reverse genetics systems. These findings highlight a critical limitation in the vRNA incorporation mechanism of influenza C virus, contrasting with that of influenza A virus. Hence, further studies are required to elucidate specific vRNA packaging signals and optimize vRNA expression levels to improve the production of infectious influenza C virus particles.

## 1. Introduction

The influenza C virus genome comprises seven RNA segments (PB2, PB1, P3, HEF, NP, M, and NS) with negative polarity [1]. Several reverse genetics studies on the influenza C virus have been conducted [2,3,4]. Previously, we generated recombinant influenza C/Ann Arbor/1/50 (C/AA/50) by transfecting 16 or 11 plasmid DNAs into 293T cells [3]: seven Pol I plasmids for virus RNA (vRNA) expression and nine or four plasmids for virus protein expression. In this study, we observed that the infectious titer of the transfected 293T cell supernatant ranged from 10^1^ to 10^3^ of the 50% egg infectious dose (EID)_50_, although the hemagglutination (HA) titer of the supernatant was as high as 1:4 to 1:8 [3]. This finding strongly suggests that the supernatant of the plasmid-transfected 293T cells contained a higher number of non-infectious influenza C virus particles. Furthermore, this result contrasts with the findings for influenza A virus; in a reverse genetics system, influenza A virus particles possessing a full set of vRNA segments (eight segments) were the most efficiently generated from plasmid-transfected 293T cells [5,6,7].

However, we reported a higher generation efficiency of influenza C virus-like particles (C-VLPs) containing specific vRNA segments. For instance, C-VLPs containing GFP-vRNAs could be recovered efficiently (10^6^ C-VLPs/mL) [8], and C-VLPs possessing a given vRNA segment (PB2-, PB1-, P3-, HEF-, NP-, M-, or NS-vRNA) were generated as efficiently as those containing GFP-vRNA [3]. Thus, it is likely that the C-VLPs comprising a given vRNA segment were efficiently generated.

**Figure 1:**
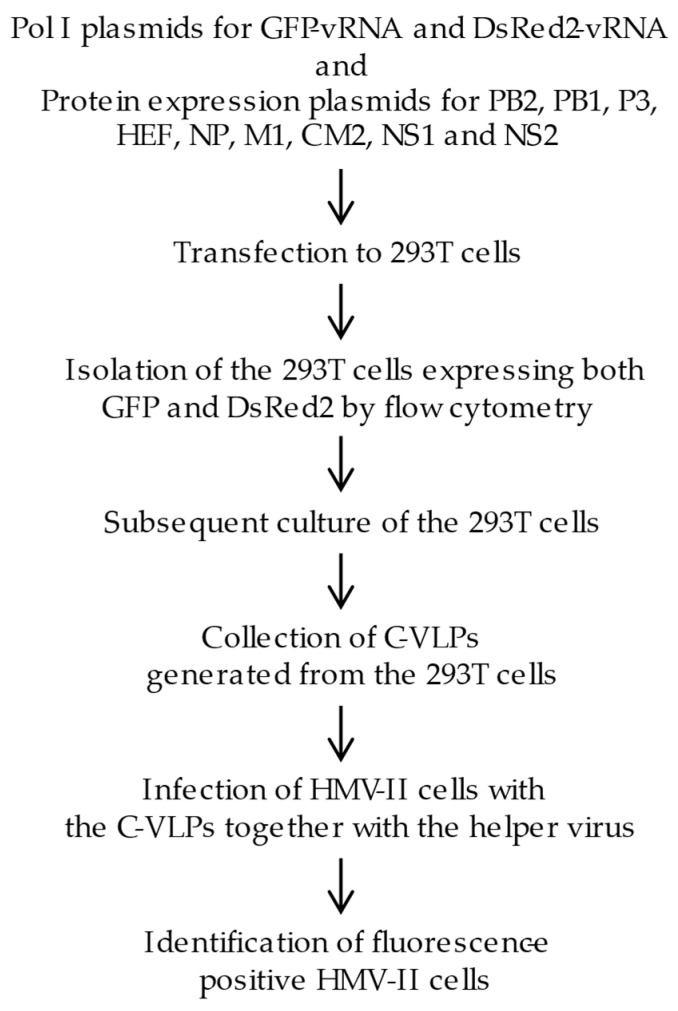
A flow chart of the experimental design. Eleven plasmids expressing reporter gene RNA genomes (GFP-vRNA and DsRed2-vRNA) and virus proteins (PB2, PB1, P3, HEF, NP, M1, CM2, NS1, and NS2) were transfected into 293T cells. In these cells, GFP-vRNA and DsRed2-vRNA s (each flanked by the non-coding regions of Segments 5 and 6, respectively) were replicated and transcribed by the RNA polymerase complex comprising the PB2, PB1, P3, and NP proteins, resulting in the expression of GFP and DsRed2 proteins. At 48 h post transfection, the cells were subjected to flow cytometry, and cells expressing both GFP and DsRed2 were sorted. The sorted cells were then incubated for 48 h, and the influenza C virus-like particles (C-VLPs) generated from the cells were collected. HMV-II cells were infected with these C-VLPs, followed by superinfection with a helper virus (C/Ann Arbor/1/50), and the fluorescence-positive cells were examined.

Based on these findings, we hypothesized that the reduced efficiency of generating infectious influenza C viruses in a reverse genetics system is due to the inefficient incorporation of two or more vRNA segments into one particle [3,9]. In the present study, we generated C-VLPs from 293T cells expressing both GFP- and DsRed2-vRNAs, each flanked by the non-coding regions (NCRs) of Segments 5 and 6, respectively. This was based on the hypothesis that NCRs derived from adjacent vRNA segments are more likely to associate efficiently during packaging. We further examined whether the C-VLPs contained GFP- or DsRed2-vRNAs by detecting fluorescence in the C-VLP-infected cells (Figure 1).

## 2. Materials and Methods

### 2.1. Cells and Viruses

293T cells were maintained in Dulbecco’s modified Eagle’s medium with 10% fetal bovine serum (FBS) [8]. HMV-II cells were maintained in RPMI 1640 medium supplemented with 10% calf serum [10]. Influenza C virus (C/AA/50) was grown in the amniotic cavity of 9-day-old embryonated chicken eggs, as described previously [11].

### 2.2. Plasmids

Two types of Pol I plasmids were constructed for vRNA expression in transfected 293T cells. The pPolI/NP-AA.GFP(−) construct was designed by inserting *GFP* cDNA, flanked by the non-coding region (NCR) sequences of the C/AA/50 NP gene (Segment 5), in an antisense orientation between the RNA polymerase I promoter and the terminator of the vector pHH21, as described previously [8]. For the pPolI/M-AA.DsRed2(−) construct, *DsRed2* was PCR-amplified using a pair of primers containing the sequences of the M gene (Segment 6) NCRs and BsmBI sites. The PCR product was digested with BsmBI and inserted in an antisense orientation between the RNA polymerase I promoter and the terminator of pHH21. The nucleotide sequences of primers and PCR protocols are available from the corresponding author upon reasonable request. The following nine plasmids for the expression of influenza C virus proteins (PB2, PB1, P3, HEF, NP, M1, CM2, NS1, and NS2) were used, as described previously [8]: pcDNA/PB2-AA, pcDNA/PB1-AA, pcDNA/P3-AA, pCAGGS.MCS/NP-AA, pME18S/HEF-AA, pCAGGS.MCS/M1-AA, pME18S/Met-CM2-YA, pME18S/NS1-YA, and pME18S/NS2-YA.

### 2.3. Generation of C-VLPs from 293T Cells Expressing a Single Type of vRNA

C-VLPs containing GFP-vRNA or DsRed2-vRNA were generated, as previously described [8,12,13,14]. Briefly, 1.0 × 10^6^ 293T cells in a 35 mm Petri dish were transfected with the following plasmids using the transfection reagent Trans-IT-LT-1 (Mirus, Madison, WI, USA): pPolI/NP-AA.GFP(−) or pPolI/ M-AA.DsRed2(−), pcDNA/PB2-AA, pcDNA/PB1-AA, pcDNA/P3-AA, pCAGGS.MCS/NP-AA, pME18S/HEF-AA, pCAGGS.MCS/M1-AA, pME18S/Met-CM2-YA, pME18S/NS1-YA, and pME18S/NS2-YA. The culture media of the transfected cells were collected 48 h post transfection (p.t.), clarified via low-speed centrifugation, and used for the infection of HMV-II cells (see Section 2.4 below).

### 2.4. Generation of VLPs from 293T Cells Expressing Two Types of vRNAs

In this experiment, 1.0 × 10^6^ 293T cells in a 35 mm Petri dish were transfected with pPolI/NP-AA.GFP(−) and pPolI/M-AA. DsRed2(−) along with the nine protein-expressing plasmids, described in Section 2.3. At 48 h p.t., the transfected 293T cells were trypsinized using Accutase (Innovative Cell Technologies, Inc., San Diego, CA, USA), re-suspended in PBS containing 3% FBS to a density of 1.0 × 10^6^ cells/mL, and then subjected to flow cytometry using a BD FACSAria Cell Sorter (Becton Dickinson, San Jose, CA, USA). Cells expressing both GFP and DsRed2 were sorted into a sterile tube, washed once with Opti-MEM (Life Technologies, Carlsbad, CA, USA), and then seeded into a 24-well plate (Nunc, Carlsbad, CA, USA), followed by incubation at 33 °C for 48 h. The culture media of the sorted cells were collected, clarified, and used to infect HMV-II cells.

### 2.5. Infection of HMV-II Cells with C-VLPs

The supernatant of the 293T cells containing C-VLPs was treated with TPCK (tosyl phenylalanyl chloromethyl ketone)-treated trypsin (20 μg/mL) at 37 °C for 10 min, followed by the addition of soybean trypsin inhibitor. The supernatant was added to an HMV-II cell monolayer, followed by incubation at 33 °C for 60 min. The cells were subsequently infected with the helper virus (C/AA/50) at a multiplicity of infection (MOI) of 5 and then incubated at 33 °C for 48 h. GFP- or DsRed2-positive HMV-II cells were observed and imaged using a fluorescence microscope (Leica Microsystems GmbH, Wetzlar, Germany).

### 2.6. Reverse-Transcription (RT)-PCR of vRNA in 293T Cells

RNA was extracted from sorted 293T cells, as described previously [3]. The extracted RNA was treated with DNase I and then reverse-transcribed using a primer complementary to the 12 nucleotides of the 3′ end of the influenza C vRNA [15]. The resulting cDNA was PCR-amplified using a set of primers specific to the GFP or DsRed2 gene sequences. The nucleotide sequences of the primers and the PCR protocols can be provided upon reasonable request.

### 2.7. Statistical Analysis

Statistical analyses were performed using GraphPad Prism version 10.2.3 (GraphPad Software, Inc., Boston, MA, USA). The data between the groups were analyzed using a paired *t*-test. Statistical significance was set at *p* < 0.05.

## 3. Results

### 3.1. Generation of C-VLPs Containing DsRed2-vRNA

Previously, we generated C-VLPs containing GFP-vRNA by transfecting 293T cells with pPolI/NP-AA.GFP(−) [8]. In the present study, we generated C-VLPs containing DsRed2-vRNA following a procedure similar to that described for pPolI/NP-AA.GFP(−) [8], by transfecting a pPol I/M-AA.DsRed2(−) plasmid into 293T cells along with nine virus protein-expressing plasmids. At 48 h p.t., the 293T cell supernatant containing C-VLPs was treated with TPCK-treated trypsin and added to HMV-II cell monolayers, followed by superinfection with a helper virus (C/AA/50). Approximately 10^6^ C-VLPs/mL were present in the supernatant of the transfected 293T cells at 48 h p.t., based on the quantification of DsRed2-positive HMV-II cells. Thus, the number of C-VLPs containing DsRed2-vRNA was comparable to that of C-VLPs containing GFP-vRNA [8], indicating that C-VLP generation efficiency remained unaffected by the reporter gene or the nucleotide sequences of NCRs flanking the reporter gene.

### 3.2. Isolation of 293T Cells Expressing Both GFP- and DsRed2-vRNAs

**Figure 2:**
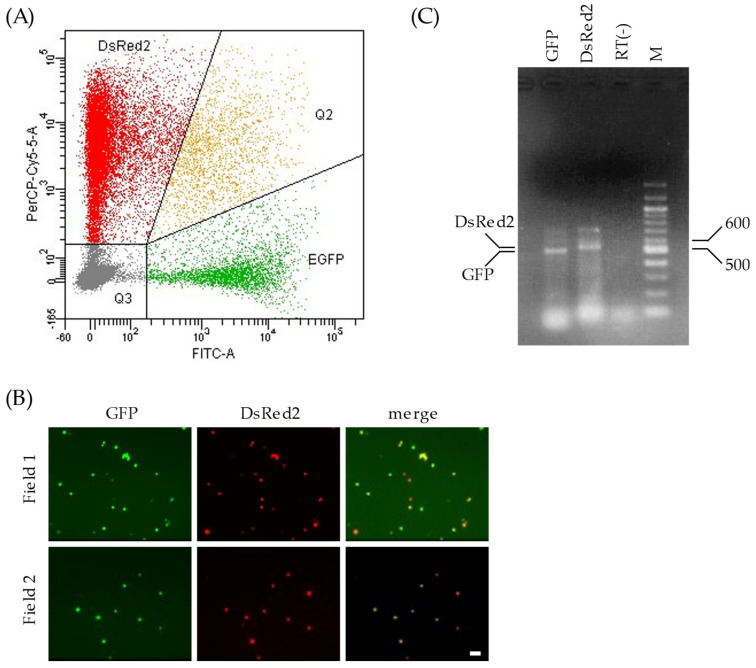
Gene expressions of plasmid-transfected 293T cells. (**A**) The transfected 293T cells were subjected to flow cytometry 48 h post transfection. The x- and y-axes in the graph indicate the intensities of GFP and DsRed2, respectively. Each 293T cell analyzed is expressed as a dot. The graph area is divided into four regions according to the proteins expressed: DsRed2, cells expressing DsRed2 alone; EGFP, cells expressing GFP alone; Q2, cells expressing both GFP and DsRed2; and Q3, cells expressing neither GFP nor DsRed2. (**B**) The sorted 293T cells were observed under a fluorescence microscope to detect GFP and DsRed2 expression. Two independent fields are shown (fields 1 and 2). Scale bar, 20 µm. (**C**) Total RNA extracted from the sorted 293T cells was treated with DNase I, reverse-transcribed, and PCR-amplified with a primer set specific to *GFP* or *DsRed2*. The cDNA preparation setup without reverse transcription was PCR-amplified using primers specific to *GFP* and electrophoresed in the lane RT(−). The main products in the corresponding lanes are shown (DsRed2, 537 bp; GFP, 504 bp). M: DNA size marker (bp).

Two Pol I plasmids, pPolI/NP-AA.GFP(−) and pPolI/M-AA.DsRed2(−), were transfected into 293T cells along with the nine expression plasmids for virus proteins, as described above. We found that 24.1% of the living cells expressed fluorescent proteins (GFP and/or DsRed2), and 3.1% of the fluorescence-positive cells expressed both GFP and DsRed2 (Figure 2A). Thus, 293T cells expressing both GFP-vRNA and DsRed2-vRNA were generated.

### 3.3. Infection of HMV-II Cells with the C-VLPs Generated from 293T Cells Expressing Both GFP- and DsRed2-vRNAs

To obtain C-VLPs possessing both GFP-vRNA and DsRed2-vRNA, we isolated transfected 293T cells using flow cytometry. 293T cells expressing both GFP and DsRed2 were sorted into sterile tubes containing Opti-MEM using a FACSAria system, according to the manufacturer’s instructions. An additional analysis using fluorescence microscopy and flow cytometry revealed that over 98% of the sorted cells expressed both GFP and DsRed2 (Figure 2B), indicating the presence of a single population of 293T cells expressing both GFP and DsRed2.

To confirm that GFP- and DsRed2-vRNAs were expressed in the sorted 293T cells, total RNA was extracted from the cells and subjected to RT-PCR using primers specific to the *GFP* or *DsRed2* sequences. The amount of PCR product of *GFP* was virtually equivalent to that of *DsRed2* (Figure 2C), suggesting that both GFP- and DsRed2-vRNAs were expressed at comparable levels in the sorted cells.

**Figure 3:**
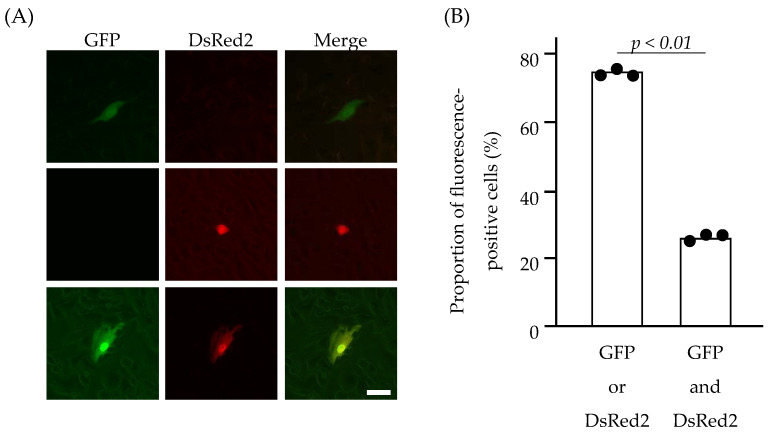
HMV-II cells infected with C-VLPs. The culture supernatant of the sorted 293T cells was added to the HMV-II monolayers, followed by superinfection with the helper virus. At 48 h post infection, the cells were observed under a fluorescence microscope (**A**). Cells expressing GFP alone, DsRed2 alone, and both GFP and DsRed2 are shown in the upper, middle, and lower panels, respectively. Merged images are shown on the right side. Scale bar, 20 µm. (**B**) The proportion of fluorescence-positive HMV-II cells expressing GFP or DsRed2 (left bar), or GFP and DsRed2 (right bar). Data obtained from three independent experiments are shown. Comparisons between groups were statistically evaluated using paired *t*-tests.

The sorted 293T cells were then seeded into the wells of a 24-well plate to produce C-VLPs into the culture medium. The culture medium was collected and clarified via low-speed centrifugation. The resulting supernatant was treated with TPCK-trypsin and a soybean trypsin inhibitor and added to HMV-II cell monolayers, followed by superinfection with the helper virus (C/AA/50). Following the incubation of HMV-II cells at 33 °C for 48 h, fluorescence-positive HMV-II cells were observed using a fluorescence microscopy (Figure 3A and Figure S1). Approximately 1% of HMV-II cells were fluorescence-positive. This finding strongly suggests that, upon infection of HMV-II cells with C-VLPs using this system, the MOI was lower than 1, and the presence of HMV-II cells expressing both GFP and DsRed2 resulted from a single infection with C-VLPs possessing both GFP-vRNA and DsRed2-vRNA.

Three independent experiments were then conducted, wherein 1 × 10^5^–2 × 10^5^ 293T cells expressing both GFP and DsRed2 were sorted and incubated to generate C-VLPs, and the entire volume of the resulting culture supernatant was added to HMV-II cell monolayers. Based on the number of fluorescence-positive HMV-II cells, an average of 0.4 C-VLP was produced from each 293T cell sorted. Furthermore, the proportion of HMV-II cells expressing both GFP and DsRed2 was 24.6%, and that expressing either GFP or DsRed2 was 75.4% (Figure 3B), which was significantly different (*p* = 0.0006). This result indicates that C-VLPs possessing both GFP- and DsRed2-vRNA were not predominant in the population of C-VLPs generated from the sorted 293T cells.

## 4. Discussion

We previously reported the inefficient generation of a recombinant influenza C virus in a reverse genetics system [3], although we adopted a system similar to that used for influenza A virus [16]. In the present study, we examined the efficiency of genome packaging for the influenza C virus using a C-VLP generation system. Our results showed that the simultaneous packaging of two different vRNA segments into a single C-VLP occurs inefficiently. This finding may explain one of the reasons for the inefficient production of infectious influenza C virus by reverse genetics.

Previous studies have reported the generation efficiency of recombinant influenza A viruses using a reverse genetics system. Fujii et al. reported that influenza A virus particles containing seven or six segments are generated less efficiently than those containing eight vRNA segments [5]. Gao et al. generated a seven-segment influenza A virus and demonstrated that efficient vRNA packaging into influenza A viruses requires the presence of eight vRNA segments [6]. Neumann et al. showed that many influenza A virus-like particles containing eight vRNA segments were generated from 293T cells [7]. Taken together, these findings indicate that, in a reverse genetics system, influenza A virus particles possessing a full set of vRNA segments (eight segments) were generated most efficiently from plasmid-transfected 293T cells.

In contrast to the influenza A virus, the generation efficiency of the influenza C virus in a reverse genetics system is insufficient [3]. In this system, one in approximately 10^3^ 293T cells will likely express a full set of vRNA segments (seven segments), as reported for a full set of vRNA segments (eight segments) of the influenza A virus system (one in 10^2.8^–10^3.3^ 293T cells) [16]. However, the ratio of the infectious titer to the HA titer in the culture supernatant was extremely low (up to 10^2^), indicating the presence of a large number of non-infectious influenza C viruses in the supernatant of plasmid-transfected cells [3]. In contrast, in our VLP generation system, the supernatant of transfected 293T cells exhibited a 1:8 HA titer and contained 10^6^ C-VLPs/mL [8]. Taken together, these findings strongly suggest that influenza C virus particles with multiple (more than one type) segments are produced less efficiently, resulting in less efficient production of particles with a full set of segments.

In the present study, C-VLPs produced from cells expressing two different genes were analyzed to test the hypothesis that multiple vRNAs are incorporated into a single particle of the influenza C virus inefficiently. Transfected 293T cells expressing both GFP- and DsRed2-vRNAs were sorted by flow cytometry and cultured. The proportion of C-VLPs possessing both GFP- and DsRed2-vRNAs was examined by adding the 293T supernatant to HMV-II cells, followed by microscopic analysis. The results revealed that C-VLPs possessing both GFP- and DsRed2-vRNAs were not predominant in the C-VLP population generated from sorted 293T cells (Figure 3B), although the expression levels of GFP- and DsRed2-vRNAs in the sorted cells were almost comparable (Figure 2C). This finding indicates that the simultaneous incorporation of GFP- and DsRed2-vRNAs into a single C-VLP is inefficient, at least under this condition. Collectively, these findings suggest that the reduced efficiency of incorporating more than two vRNA segments into one particle could contribute to the lower generation efficiency of the infectious influenza C viruses in a reverse genetics system.

This study had several limitations, and enhancing the current method could yield a more accurate proportion of C-VLPs possessing both GFP- and DsRed2-vRNAs. The incorporation efficiency of the two reporter vRNAs may be more accurately assessed in the presence of five other vRNA segments. The packaging sequences have been reported for influenza A [17,18,19,20,21] and influenza B [22] viruses. Although the involvement of NCR sequences in replication has been reported, the packaging sequences of the influenza C virus remain unknown [4,23,24]. Therefore, reporter gene cDNAs flanked by authentic packaging signal sequences (consisting of the NCRs of Segment 5 and 6 followed by the potential packaging signal sequences located at both ends of the coding region of Segment 5 and 6) would also be useful for elucidating a more accurate proportion of C-VLPs. Furthermore, the precise quantification of vRNAs in transfected cells could be improved. For example, vRNA quantification using a digital PCR method would be useful in assessing the proportion of C-VLPs possessing reporter gene vRNAs. Ishida et al. produced recombinant viruses efficiently using an influenza D virus reverse genetics system by adjusting the amount of each plasmid to be transfected [25]. Therefore, it may be necessary to adjust the amount of the plasmid to create more infectious particles for the influenza C virus efficiently.

## 5. Conclusions

Incorporating more than two vRNA segments into a single C-VLP is an inefficient process. This observation accounts for the inefficient generation of infectious influenza C viruses using the reverse genetics system.

## Data Availability

Data can be provided upon reasonable request.

## Supplementary Materials

The following supporting information can be downloaded at: https://www.mdpi.com/article/10.3390/v17030350/s1, Figure S1: Images of HMV-II cells infected with influenza C virus-like particles (C-VLPs).

## Abbreviations

The following abbreviations are used in this manuscript:

C-VLP	Influenza C virus-like particle
MOI	Multiplicity of infection
NCR	Non-coding region
vRNA	Virus ribonucleic acid
