# Supplementary material for: Simultaneous Packaging of Two Different RNA Segments into an Influenza C Virus-like Particle Occurs Inefficiently
Source: Viruses. 2025 Feb 28;17(3):350. doi: 10.3390/v17030350 (PMC11946231; doi:10.3390/v17030350)
Supplement: Supplementary file 1 [file viruses-17-00350-s001.zip › viruses-3485697-Figure S1 R.pdf]

Simultaneous packaging of two different RNA segments into an influenza C virus-like particle occurs inefficiently

Supplementary Figure

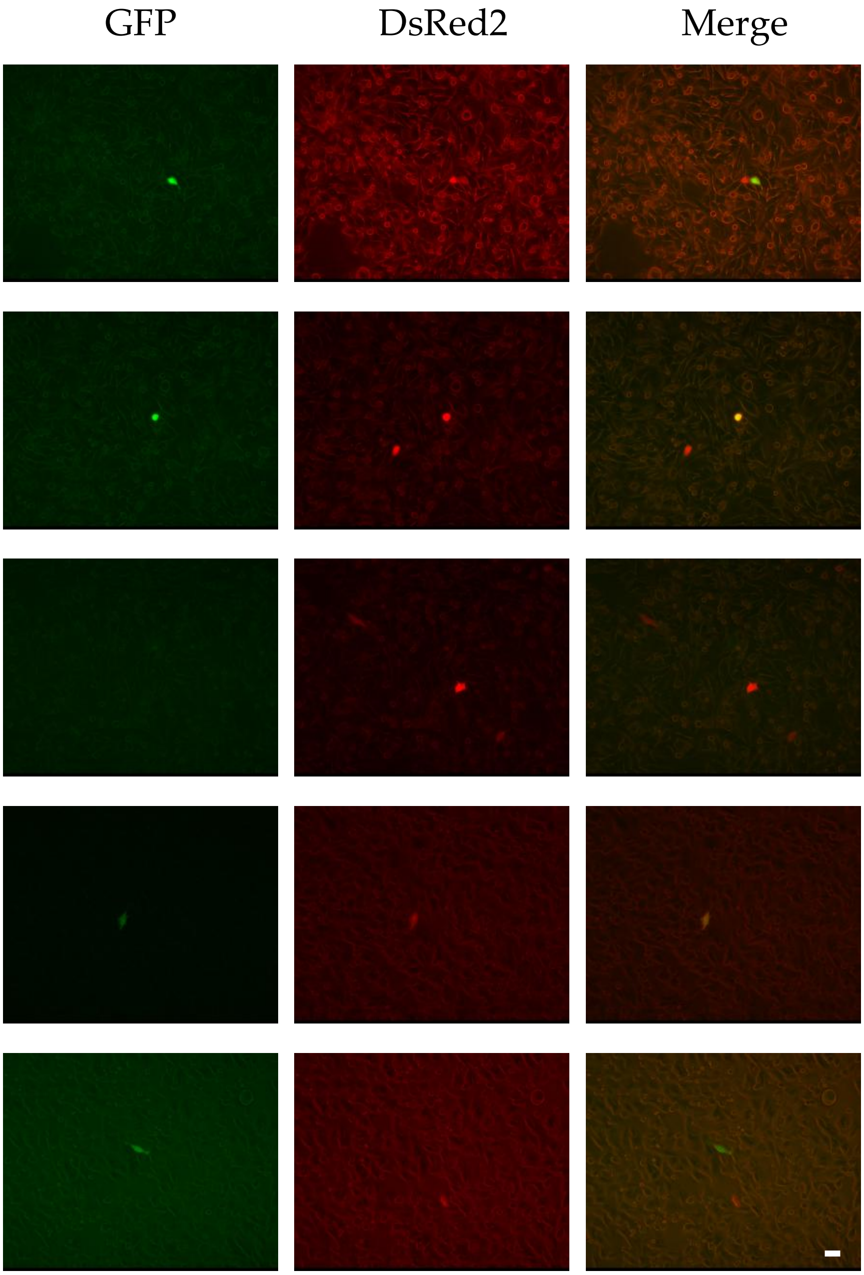

Figure S1. Images of HMV-II cells infected with influenza C virus-like particles (C-VLPs). C-VLP-infected HMV-II cells were observed under a fluorescence microscope. Cells expressing GFP and DsRed2 are displayed in the left and middle panels, respectively. The merged images are presented in the right panels. Cells other than those indicated in Fig. 3(A) are shown. Scale bar, 20 μm.
